# Novel Regulatory Roles of Hydrogen Sulfide in Health and Disease

**DOI:** 10.3390/biom12101372

**Published:** 2022-09-25

**Authors:** Csaba Szabo

**Affiliations:** Chair of Pharmacology, Section of Medicine, University of Fribourg, CH-1700 Fribourg, Switzerland; csaba.szabo@unifr.ch

**Keywords:** hydrogen sulfide, gasotransmitters, cell biology, neuroscience, kidney, mitochondria, cancer

Following Prof. Hideo Kimura’s seminal discovery (1996) of its role as a neuromodulator in the brain [1], various regulatory roles of the endogenous gaseous transmitter hydrogen sulfide (H_2_S) have been discovered in mammalian cells in health and disease. These roles include—among others—various transmitter and modulator roles in the nervous system, regulatory roles in the cardiovascular system (modulation of vascular tone, angiogenesis, and cardiac function), modulatory effects on various aspects of cellular metabolism, and regulatory roles in a multitude of conditions in health and disease. The physiological roles and the pharmacological modulation of H_2_S have been summarized in two recent, comprehensive review articles [2,3], while various pathophysiological aspects of H_2_S are covered in various specialized review articles, e.g., [4,5,6,7,8,9,10].

The goal of this Special Issue was to honor the 25th anniversary of the publication of Prof. Kimura’s landmark paper, and to showcase some of the most recent developments in the field of H_2_S biology. Professor Kimura himself has prepared a recent update on H_2_S and polysulfide signalling, with particular emphasis on its roles in the central nervous system—a field that was initiated by him and where his group has made multiple seminal contributions over the last 25 years [11]. This Special Issue was also accompanied by the publication of a mini-interview with Prof. Kimura to highlight the circumstances of the initial discovery 25 years ago and his perspective on the subsequent expansion of the H_2_S/polysulfide field (https://blog.mdpi.com/2021/02/15/4425-revision-v1; accessed on 20 September 2022.

This Special Issue featured several additional review articles focusing on various specialized aspects of H_2_S biology. With respect to pathophysiological aspects, Beck and Pfeilschifter summarized the current state-of-the-art on the role of H_2_S—on its own, as well as in concert with other gasotransmitters such as nitric oxide and carbon monoxide—in the modulation of renal glomerular diseases [12]. In addition, Frederic Bouillaud has written a review on the bioenergetic aspects of H_2_S, with focus on sulfide oxidation [13]. With respect to methodological aspects, Echizen, Sasaki and Hanaoka summarized some of the current methods and techniques for the detection of H_2_S and related molecules, using, among others, LC/MS, Raman imaging and fluorescent probes [14]. In the emerging field of therapeutic applications of H_2_S biology, Merz and colleagues from the University of Köln have written an article on the potential therapeutic applications of sodium thiosulfate—a clinically approved compound—which serves, among other pharmacological actions, as a slow-releasing H_2_S donor and may be clinically useful in various pathophysiological conditions associated with H_2_S deficiency [15]. On a related subject, Piragine and colleagues from the University of Pisa have written an article on natural H_2_S donors (such as allyl sulfide compounds and organic isothiocyanates) as potential therapeutic agents in hypertension [16]. A common theme between these two review articles is that both approaches seek to expedite clinical application of H_2_S without engaging the standard (and lengthy and—for most academic groups—prohibitively expensive) full-scale ‘original drug development approach’: in the case of thiosulfate, through the drug repurposing/repositioning approach; in the case of natural compounds, through the potential utilization of natural supplements.

With respect to original articles, the wide variety of approaches and model systems (ranging from lower organisms such as Drosophila to murine models and human cell systems), and the wide variety of disease conditions (from hypertension to kidney disease and colon cancer) covered in the Special Issue further illustrates the wide-ranging roles and functions of H_2_S in health and disease. Regarding the physiological regulatory aspects of H_2_S in the central nervous system, Zatsepina and colleagues delineated the genes involved in the regulation of H_2_S homeostasis in Drosophila in the context of learning and memory formation [17]. It should be mentioned that the original article of Prof. Kimura in 1996 has already predicted that H_2_S might have such roles in the central nervous system; this prediction has since been confirmed in multiple models and experimental systems [3].

Two original articles published in this Special Issue focused on the actions of mitochondrially targeted H_2_S. da Costa Marques and colleagues demonstrate the vasorelaxant effects of the mitochondrially targeted H_2_S donor, AP39, in mouse mesenteric rings [18], while Juriasingani and colleagues demonstrate the beneficial effect of the same compound in a renal graft reperfusion model [19]. Since the initial description and characterization of this donor in 2014 [20,21], this compound has been used in a wide variety of models and systems, either as a vasorelaxant or as a cytoprotective agent. The fact that it targets H_2_S to the mitochondria can be also considered in the context of the review of Prof. Bouillaud [13] already mentioned above.

Several studies that appear in this Special Issue utilized H_2_S salts such as NaHS or Na_2_S, which are often referred to in the literature as “fast-acting H_2_S donors”, although this terminology (as well as the actual delivery of H_2_S to biological systems performed this way, which creates high peak concentrations and is not a good way to mimic the endogenous H_2_S biosynthesis) is not without problems, as discussed in recent reviews [2,3]. Nevertheless, using this pharmacological approach, various protective and beneficial effects of H_2_S have been highlighted in this Special Issue, such as improved insulin signalling in the adipose tissue [22] and protective effects in a model of kidney ischemia-reperfusion [23].

Finally, a group of articles published in this Special Issue focused on the role of H_2_S in various forms of cancer. Since the demonstration [24] that the H_2_S-producing enzyme CBS is overexpressed in colon cancer, and that it plays various cancer-cell-supporting roles (such as cytoprotection, proliferative effects, pro-angiogenic effects and others), this field has steadily expanded (as reviewed recently in [10]). In the current Special Issue, Wrobel’s group published two articles focusing on the expression patterns of various H_2_S-producing enzymes in a variety of commonly used cell lines, and identified some commonalities as well as important cell-type-differences [25,26]. Moreover, in a study utilizing a patient-derived xenograft model, our own group has demonstrated that those colon cancers that express higher levels of CBS, when implanted onto nude mice, proliferate faster and respond better to pharmacological CBS inhibition than those colon cancers that express low levels of CBS [27]. These observations—together with several other lines of recently emerging data regarding the importance of H_2_S overproduction in various cancers [28,29,30]—may help with the future clinical translation of the CBS inhibition concept for the experimental therapy of cancer.

## References

[1] Abe K., Kimura H. (1996). The possible role of hydrogen sulfide as an endogenous neuromodulator. J. Neurosci..

[2] Szabo C., Papapetropoulos A. (2017). International Union of Basic and Clinical Pharmacology. CII: Pharmacological modulation of H_2_S levels: H_2_S donors and H_2_S biosynthesis inhibitors. Pharmacol. Rev..

[3] Cirino G., Szabo C., Papapetropoulos A. (2022). Physiological roles of hydrogen sulfide in mammalian cells, tissues and organs. Physiol. Rev..

[4] Paul B.D., Snyder S.H. (2018). Gasotransmitter hydrogen sulfide signaling in neuronal health and disease. Biochem. Pharmacol..

[5] Kimura H. (2019). Signaling by hydrogen sulfide (H_2_S) and polysulfides (H_2_Sn) in the central nervous system. Neurochem. Int..

[6] Petrovic D., Kouroussis E., Vignane T., Filipovic M.R. (2021). The role of protein persulfidation in brain aging and neurodegeneration. Front. Aging Neurosci..

[7] Pacitti D., Scotton C.J., Kumar V., Khan H., Wark P.A.B., Torregrossa R., Hansbro P.M., Whiteman M. (2021). Gasping for sulfide: A critical appraisal of hydrogen sulfide in lung disease and accelerated aging. Antioxid. Redox Signal..

[8] LaPenna K.B., Polhemus D.J., Doiron J.E., Hidalgo H.A., Li Z., Lefer D.J. (2021). Hydrogen sulfide as a potential therapy for heart failure—Past, present and future. Antioxidants.

[9] Scammahorn J.J., Nguyen I.T.N., Bos E.M., Van Goor H., Joles J.A. (2021). Fighting oxidative stress with sulfur: Hydrogen sulfide in the renal and cardiovascular systems. Antioxidants.

[10] Ascenção K., Szabo C. (2022). Emerging roles of cystathionine β-synthase in various forms of cancer. Redox. Biol..

[11] Kimura H. (2021). Hydrogen sulfide (H_2_S) and polysulfide (H_2_Sn) signaling: The first 25 years. Biomolecules.

[12] Beck K.F., Pfeilschifter J. (2022). The pathophysiology of H_2_S in renal glomerular diseases. Biomolecules.

[13] Bouillaud F. (2022). Sulfide oxidation evidences the immediate cellular response to a decrease in the mitochondrial ATP/O_2_ Ratio. Biomolecules.

[14] Echizen H., Sasaki E., Hanaoka K. (2021). Recent advances in detection, isolation, and imaging techniques for sulfane sulfur-containing biomolecules. Biomolecules.

[15] Merz T., McCook O., Brucker C., Waller C., Calzia E., Radermacher P., Datzmann T. (2022). H_2_S in critical illness—A new horizon for sodium thiosulfate?. Biomolecules.

[16] Piragine E., Citi V., Lawson K., Calderone V., Martelli A. (2022). Potential effects of natural H_2_S-donors in hypertension management. Biomolecules.

[17] Zatsepina O.G., Chuvakova L.N., Nikitina E.A., Rezvykh A.P., Zakluta A.S., Sarantseva S.V., Surina N.V., Ksenofontov A.L., Baratova L.A., Shilova V.Y. (2022). Genes responsible for H_2_S production and metabolism are involved in learning and memory in Drosophila melanogaster. Biomolecules.

[18] da Costa Marques L.A., Teixeira S.A., de Jesus F.N., Wood M.E., Torregrossa R., Whiteman M., Costa S.K.P., Muscará M.N. (2022). Vasorelaxant activity of AP39, a mitochondria-targeted H_2_S donor, on mouse mesenteric artery rings in vitro. Biomolecules.

[19] Juriasingani S., Ruthirakanthan A., Richard-Mohamed M., Akbari M., Aquil S., Patel S., Al-Ogaili R., Whiteman M., Luke P., Sener A. (2021). Subnormothermic perfusion with H_2_S donor AP39 improves DCD porcine renal graft outcomes in an ex vivo model of kidney preservation and reperfusion. Biomolecules.

[20] Le Trionnaire S., Perry A., Szczesny B., Szabo C., Winyard P.G., Whatmore J.L., Wood M.E., Whiteman M. (2014). The synthesis and functional evaluation of a mitochondria-targeted hydrogen sulfide donor, (10-oxo-10-(4-(3-thioxo-3H-1,2-dithiol-5-yl)phenoxy)decyl) triphenylphosphonium bromide (AP39). Med. Chem. Commun..

[21] Szczesny B., Módis K., Yanagi K., Coletta C., Le Trionnaire S., Perry A., Wood M.E., Whiteman M., Szabo C. (2014). AP39, a novel mitochondria-targeted hydrogen sulfide donor, stimulates cellular bioenergetics, exerts cytoprotective effects and protects against the loss of mitochondrial DNA integrity in oxidatively stressed endothelial cells in vitro. Nitric Oxide.

[22] Kowalczyk-Bołtuć J., Wiórkowski K., Bełtowski J. (2022). Effect of exogenous hydrogen sulfide and polysulfide donors on insulin sensitivity of the adipose tissue. Biomolecules.

[23] Hashmi S.F., Rathore H.A., Sattar M.A., Johns E.J., Gan C.Y., Chia T.Y., Ahmad A. (2021). Hydrogen sulphide treatment prevents renal ischemia-reperfusion injury by inhibiting the expression of ICAM-1 and NF-kB concentration in normotensive and hypertensive rats. Biomolecules.

[24] Szabo C., Coletta C., Chao C., Módis K., Szczesny B., Papapetropoulos A., Hellmich M.R. (2013). Tumor-derived hydrogen sulfide, produced by cystathionine-β-synthase, stimulates bioenergetics, cell proliferation, and angiogenesis in colon cancer. Proc. Natl. Acad. Sci. USA.

[25] Kaczor-Kamińska M., Kaminski K., Wróbel M. (2021). The expression and activity of rhodanese, 3-mercaptopyruvate sulfurtransferase, cystathionine γ-lyase in the most frequently chosen cellular research models. Biomolecules.

[26] Jurkowska H., Wróbel M., Jasek-Gajda E., Rydz L. (2022). Sulfurtransferases and cystathionine beta-synthase expression in different human leukemia cell lines. Biomolecules.

[27] Hellmich M.R., Chao C., Módis K., Ding Y., Zatarain J.R., Thanki K., Maskey M., Druzhyna N., Untereiner A.A., Ahmad A. (2021). Efficacy of novel aminooxyacetic acid prodrugs in colon cancer models: Towards clinical translation of the cystathionine β-synthase inhibition concept. Biomolecules.

[28] Erdélyi K., Ditrói T., Johansson H.J., Czikora Á., Balog N., Silwal-Pandit L., Ida T., Olasz J., Hajdú D., Mátrai Z. (2021). Reprogrammed transsulfuration promotes basal-like breast tumor progression via realigning cellular cysteine persulfidation. Proc. Natl. Acad. Sci. USA.

[29] Khan N.H., Wang D., Wang W., Shahid M., Khattak S., Ngowi E.E., Sarfraz M., Ji X.Y., Zhang C.Y., Wu D.D. (2022). Pharmacological inhibition of endogenous hydrogen sulfide attenuates breast cancer progression. Molecules.

[30] Ascenção K., Dilek N., Zuhra K., Módis K., Sato T., Szabo C. (2022). Sequential accumulation of ‘driver’ pathway mutations induces the upregulation of hydrogen-sulfide-producing enzymes in human colonic epithelial cell organoids. Antioxidants.

